# Practice guidelines for therapeutic drug monitoring of voriconazole: a consensus review of the Japanese Society of Chemotherapy and the Japanese Society of Therapeutic Drug Monitoring

**DOI:** 10.1007/s10156-013-0607-8

**Published:** 2013-05-15

**Authors:** Yukihiro Hamada, Issei Tokimatsu, Hiroshige Mikamo, Masao Kimura, Masafumi Seki, Shunji Takakura, Norio Ohmagari, Yoshiko Takahashi, Kei Kasahara, Kazuaki Matsumoto, Kenji Okada, Masahiro Igarashi, Masahiro Kobayashi, Takahiro Mochizuki, Yoshifumi Nishi, Yusuke Tanigawara, Toshimi Kimura, Yoshio Takesue

**Affiliations:** 1 Department of Infection Control and Prevention, Aichi Medical University Graduate School of Medicine, Aichi, Japan; 2 Internal Medicine II, Oita University Faculty of Medicine, Oita, Japan; 3 Department of Pharmacy, Aichi Medical University Hospital, Aichi, Japan; 4 Division of Infection Control and Prevention, Osaka University Medical Hospital, Osaka, Japan; 5 Department of Infection Control and Prevention, Kyoto University Hospital, Kyoto, Japan; 6 Disease Control and Prevention Center, National Center for Global Health and Medicine Hospital, Tokyo, Japan; 7 Department of Pharmacy, Hyogo Medical College Hospital, Hyogo, Japan; 8 Center for Infectious Diseases, Nara Medical University, Nara, Japan; 9 Department of Clinical Pharmacy and Pharmacology, Graduate School of Medical and Dental Sciences, Kagoshima University, Kagoshima, Japan; 10 Department of Pharmacy, Tokyo Women’s Medical University Hospital, Tokyo, Japan; 11 Department of Pharmacy, Toranomon Hospital, Tokyo, Japan; 12 Department of Pharmacy, Kitasato University Hospital, Kanagawa, Japan; 13 Department of Pharmacy, Shizuoka Cancer Center, Shizuoka, Japan; 14 Department of Pharmacy, Kyorin University School of Medicine, Tokyo, Japan; 15 Department of Clinical Pharmacokinetics and Pharmacodynamics, School of Medicine, Keio University, Tokyo, Japan; 16 Department of Infection Control and Prevention, Hyogo College of Medicine, Hyogo, Japan; 17 Sectional Committee of Practice Guidelines for TDM; Antimicrobial agents, the Japanese Society of Therapeutic Drug Monitoring, Niigata, Japan; 18 Committee of Practice Guidelines for TDM of Antimicrobial Agents, Japanese Society of Chemotherapy, Nichinai Kaikan B1, 3-28-8 Hongo, Bunkyo-ku, Tokyo, 113-0033 Japan

**Keywords:** Guideline, Voriconazole, Therapeutic drug monitoring

## Introduction

Voriconazole (VRCZ) is a triazole antifungal developed for the treatment of fungal infectious disease and is available for both oral and intravenous administration. It has potent activity against a broad spectrum of clinically significant pathogens, including *Aspergillus*, *Candida*, *Cryptococcus*, *Fusarium*, and *Scedosporium* [1]. VRCZ has a nonlinear pharmacokinetic profile with wide inter- and intraindividual variability [2]. This variability is caused by many factors, such as sex, age, race, genotypic variation, liver dysfunction, and the presence of food. Another important factor influencing the VRCZ pharmacokinetic profile is drug–drug interactions with CYP450 inhibitors as well as inducers. Genotypic variation in the metabolizing enzyme CYP2C19 is one of the major determinants of the VRCZ blood level. The frequency of poor metabolizers (PM), with defective or diminished metabolic capacity, is higher among Japanese patients. Variability in the plasma concentrations, arising from these previously mentioned aspects, may lead to variability in efficacy or toxicity. Determining the plasma concentration is indicated in some situations to guide dosing and to individualize and improve the treatment options, resulting in a better therapeutic outcome or fewer side effects.

In this guideline, we review the factors that influence the VRCZ pharmacokinetic profile, the data supporting exposure–effect and exposure–toxicity relationships that make broad recommendations for therapeutic drug monitoring difficult for VRCZ, and provide indications for which therapeutic drug monitoring is reasonable based on currently available data (for example, in children). We also outline ways in which the existing problems can be solved.

## Methods

The practice guidelines for therapeutic drug monitoring (TDM) of VRCZ were reviewed by a practice guideline committee, consisting of 18 experts in the field of TDM (Expert Panel) convened from the Japanese Society of Chemotherapy (JSC) and the Japanese Society of Therapeutic Drug Monitoring (JSTDM). The Expert Panel reviewed papers published since 2000 and additionally analyzed data before 1999, if necessary. Computerized PUBMED searches of the English-language literature and Igaku-Chuo-Zasshi searches of the Japanese-language literature were performed using the terms “voriconazole” and “therapeutic drug monitoring” and focused mainly on human studies; however, animal studies and in vitro data were included if necessary. The committee specifically looked for existing randomized clinical trials and existing meta-analysis using a general database (i.e., MEDLINE, EMBASE), and the Cochrane Library (both The Cochrane Database of Systematic Reviews and Database of Abstracts of Reviews of Effectiveness); however, few randomized clinical trials and meta-analyses were available and many recommendations were developed from observational studies or small case studies.

To evaluate evidence regarding TDM, the Expert Panel followed the Canadian Task Force recommendations [3], which included a systematic weighting of the quality of the evidence and a graded recommendation according to the Minds classification (Table 1). The Expert Panel met in person on 7 occasions and by E-mail via mailing lists on 533 occasions. The draft guidelines for the executive summary were placed on the homepage of the JSC and JSTDM. Feedback from external public comments was obtained between April 9, 2012, and May 8, 2012. The guidelines for the Japanese version were approved by the JSC and JSTDM Board of Directors and were published in the *Japanese Journal of Chemotherapy* in June 2012.

**Table 1:** Grading system for ranking recommendations and evidence level adopted in the guideline

Category, grade	Definition
Strength of recommendation	
A	Good evidence to support a recommendation for use
B	Moderate evidence to support a recommendation for use
C1	Recommendation for use regardless of poor evidence
C2	Poor evidence to support a recommendation for use
D	Good-to-moderate evidence to support a recommendation against use
Quality of evidence	
I	Evidence from ≥1 properly randomized, controlled trial
II	Evidence from ≥1 well-designed clinical trial, without randomization from cohort or case-controlled analytical studies, multiple time-series, or dramatic results from uncontrolled experiments.
III	Evidence from opinions of respected authorities, based on clinical experience, descriptive studies, or reports of expert committees

All the members of the practice guideline committee complied with the JSC policy on conflicts of interest, which requires the disclosure of any financial or other interest that might be construed as constituting an actual, potential, or apparent conflict. Potential conflicts of interest are listed in the Acknowledgments section. The committee will determine the need for revisions to the guidelines at 3-year intervals.

## Indications for TDM

### Executive summary

- TDM for VRCZ is recommended for patients with a poor response to therapy or suspected toxicity (B-II).
- TDM is considered in patients with a severe form of mycosis, such as invasive *Aspergillus* infection (C1-III).
- Visual adverse events may be associated with higher plasma concentrations; however, many of these events have been reported to be transient. TDM is recommended especially for patients with sustained visual disturbances (C1-III).
- VRCZ is extensively metabolized via cytochrome P450 (CYP), and significant interpatient variability in VRCZ serum concentrations because of polymorphisms has been observed. TDM is recommended for patients receiving drugs that are metabolized by CYP450 (B-II).
- TDM is considered in patients undergoing hematopoietic stem cell transplantation (HSCT) who are being treated with VRCZ as prophylaxis for deep-seated mycosis (C1-III).
- TDM is considered for pediatric patients, as their blood concentrations of VRCZ are highly variable and fluctuating (C1-III).

### Literature review

Tan et al. [4] mentioned that routine monitoring of VRCZ concentration to prevent elevated liver function and visual disturbances was unlikely to add any clinical value. This conclusion was based on the premise that regular monitoring of liver function while patients are receiving VRCZ may be sufficient and visual disturbances represent, in general, a transient and mild effect that rarely necessitates discontinuation of VRCZ therapy. In addition, lower and upper target thresholds for VRCZ have been suggested, but studies to date have not been appropriately designed or powered to reveal any definitive association with its efficacy and toxicity [5]. Therefore, routine assessment of TDM in VRCZ cannot be recommended as a mandatory maneuver except in certain circumstances in this guideline. The committee defined particular indications for TDM in several situations, such as lack of response to therapy or evidence of toxicity, in which case the selective indication for TDM is clinically useful.

VRCZ is associated with adverse events, including visual disturbance and hepatic enzyme elevation with drug overdosing. The steady-state blood concentration of VRCZ has large interpersonal variability because it can be affected by several factors, including patient age, race, drug–drug interactions, route of administration, cytochrome P450 (CYP) polymorphism (mainly CYP2C19), and race because of the polymorphism [5–12]. Several observational studies have demonstrated that the blood trough level was associated with adverse events [8, 10, 13–15].

Examination of weekly plasma concentration data from patients in ten studies suggested that patients who reported visual adverse events (VAE) had higher VRCZ plasma concentrations [12]. Kakuda et al. [16] mentioned that coadministration of etravirine with VRCZ resulted in higher etravirine exposure, with an area under the plasma concentration–time curve during 0–12 h (AUC_12h_) that was 1.36 fold larger. The VRCZ plasma concentrations were slightly raised, but no grade 3 or 4 adverse events were observed during treatment. Park et al. [17] conducted a randomized controlled trial to investigate the efficacy of TDM of VRCZ against invasive fungal infections. Although the proportion of VRCZ discontinuation due to adverse events was significantly lower in the TDM group than in the non-TDM group, routine TDM of VRCZ did not reduce the incidence of drug-related adverse events because of the early occurrence of adverse events compared with the time needed for optimizing VRCZ levels based on TDM.

Variability in the plasma concentration of VRCZ both within and between individuals is high, especially in pediatric patients. The in vitro metabolism of VRCZ by liver microsomes mirrored the in vivo clearance differences in children versus adults, with VRCZ clearance being approximately threefold higher in children than in adults [18]. The VRCZ pharmacokinetics in children are highly variable, particularly for oral formulations [19]. In an open-label study of 12 immunocompromised children, wide intra- and interindividual variations in the plasma VRCZ levels were confirmed [20]. Karlsson et al. [21] suggested that children are especially at risk because VRCZ exhibits markedly reduced oral bioavailability in children compared with adults (44.6 vs. 96 %). Driscoll et al. [22] conducted a study of children who were switched from intravenous to oral treatment. In the study, large intersubject variability was observed. In the steady state during oral treatment, children had higher average exposure than adults. In adolescents who were switched from intravenous to oral treatment, large intersubject variability was observed; however, VRCZ exposure in the majority of adolescents was comparable to that in adults [23].

Several studies have demonstrated that the blood trough level was associated with not only adverse events but also treatment response. Smith et al. [8] retrospectively researched patients treated with VRCZ for invasive aspergillosis. Classification and regression tree (CART) analysis showed a relationship (*P* < 0.025) between disease progression and drug concentration. Park et al. [17] confirmed the beneficial effect of routine TDM in a randomized, controlled trial. A clinical response was observed in 81 % of patients in the TDM group compared to 57 % in the non-TDM group, with a significant difference. Among allograft recipients receiving VRCZ for the prevention of fungal infections, 6 candidiasis cases were seen among the 43 patients with VRCZ levels ≤2 μg/mL, whereas none was seen among the 24 cases with higher levels (*P* = 0.061) [6]. Trifilio et al. [24] conducted a retrospective study of VRCZ concentrations in recipients who had undergone allogeneic HSCT and received VRCZ for the prophylaxis of invasive fungal infection. Their data suggested that VRCZ concentrations were unpredictable, despite standard dosing, and the acceptability of a concentration on one occasion could not be extrapolated to future concentrations in the same patient. Trifilio et al. [13] measured the steady-state plasma trough VRCZ level and found that VRCZ was undetectable in 15 % of the patients in their series.

## Pharmacokinetics–pharmacodynamics (PK–PD)

### Executive summary

- The free AUC/MIC ratio is a PK–PD parameter that appears to be associated with treatment efficacy (C1-III).
- The trough/MIC ratio can be used clinically in place of the free AUC/MIC ratio (B-II).
- Because of high bioavailability, similar PK to intravenous administration was obtained in patients with oral administration.
- Genotypic variation in the metabolizing enzyme CYP2C19 is one of the major determinants of toxicity. The frequency of poor metabolizers (PM), i.e., individuals with defective or diminished metabolic capacity, is higher among Asian patients.

### Literature review

In an animal experiment using a neutropenic murine model of disseminated *Candida albicans* infection, the treatment efficacy supported the use of the AUC/MIC ratio as a PK–PD parameter that was predictive of efficacy. Nonlinear regression analysis also suggested that the AUC/MIC ratio was strongly predictive of the treatment outcomes [25]. Andes [26] suggested that a free AUC/MIC value <25 was associated with a half-maximal antifungal effect in murine models of disseminated candidiasis.

Monte Carlo simulation can be used to assess the relationship between the VRCZ trough concentration/MIC ratio and the clinical response. The probability of a clinical response is near maximum when the trough/MIC ratio is between 2 and 5. A previous study suggested that the trough concentration is more clinically tractable [14]. Johnson and Kauffman [27] reported that the bioavailability of the oral formulation was >90 % when administered either 1 h before or 1 h after a meal, and similar PK to intravenous administration was obtained in patients with oral administration.

Mikus et al. [28] conducted a randomized controlled trial to investigate interactions with VRCZ. The coadministration of a potent CYP3A4 inhibitor led to higher and prolonged exposure to VRCZ that might have increased the risk of the development of adverse drug reactions on a short-term basis, particularly in CYP2C19 PM patients. In gene analysis of a Japanese population, the frequency of poor metabolizers was 18.8 % [29]. The genotype status of CYP2C19 and/or the coadministration of drugs that modulate CYP2C19 or CYP3A4 activities might influence VRCZ plasma levels. The influence of the genotype on VRCZ exposure is confounded by drug–drug and drug–disease interactions in the population; thus, no dose adjustments based on genotype are recommended [30]. In gene analysis in an Asian population, the frequency of the CYP2C19 PM was similar to that in the Japanese population [31]. In this study, subsequent studies have used a combination of phenotyping and genotyping tests and have confirmed marked interethnic variation in the frequency of the PM phenotype: there is a higher frequency of the trait in Asian (12–23 %) than Caucasian (1–6 %) or Black African (1–7.5 %) populations, and African-American and Arab populations appear to be similar to Caucasians. Kimura et al. [32] reported that the pharmacokinetics of VRCZ were comparatively unstable and that the concentration of VRCZ varied among individuals, regardless of the CYP2C19 genotype.

## Methods of TDM

### Executive summary

- Blood samples for TDM should be obtained in the steady state. Steady-state levels are achieved by the 5th to 7th day of conventional administration (B-II).
- The trough level is recommended for the measurement of TDM (B-II). Routine measurement of *C*_peak_ to calculate the AUC is not recommended (C2-III).

### Literature review

Purkins et al. [33] suggested that steady-state levels were achieved by the 5th to 6th day of multiple dosing, and Lazarus et al. [34] suggested that steady-state levels were achieved by the 4th to 7th day. The majority of these pharmacokinetic estimates are based upon single trough concentrations. It would be interesting to determine whether the use of additional monitoring time points to reflect the AUC more accurately might further enhance the strength of these concentration–outcome relationships. Because of the high bioavailability (>90 %) of the oral formulation [27], TDM can be performed using the same timing in patients receiving the oral administration.

## Target serum concentrations in TDM

### Executive summary

- A target trough level ≥1–2 μg/mL is recommended in terms of clinical efficacy (B-II).
- In patients with a trough level >4–5 μg/mL, elevated liver function test results potentially attributable to VRCZ should be considered (B-II).
- As VRCZ exhibits nonlinear pharmacokinetics, the blood concentration should be reevaluated in patients revealing unexpectedly abnormal serum levels or those with alteration of the dosage regimen (B-II).
- In patients receiving oral therapy, the TDM data should be evaluated after confirming the patient’s adherence to the treatment and timing of ingestion (C1-III).

### Literature review

Ueda et al. [6] and Trifilio et al. [7] concluded that trough VRCZ levels >2 μg/mL were associated with clinical efficacy. Smith et al. [8] found that favorable responses were observed at concentrations >2.05 μg/mL. Miyakis et al. [11] reported that successful outcomes were more likely among patients with a median trough VRCZ concentration >2.2 μg/mL.

Pascual et al. [9] reported that a lack of response to therapy was more frequent among patients with VRCZ ≤1 μg/mL than in patients with VRCZ >1 μg/mL. Okuda et al. [10] found a significant difference (*P* < 0.05) in average trough blood concentrations between patients in whom VRCZ was effective and those in whom VRCZ was ineffective (8.21 ± 2.19 vs. 1.01 ± 0.86 μg/mL). Hamada et al. [12] reported that for graded cutoff values within the range of 1.0–3.0 μg/mL, VRCZ >1.0 μg/mL was used as the VRCZ trough blood concentration (*P* < 0.0001). Because of the small number of PK–PD analyses concerning clinical efficacy in each study, target serum concentrations were not demonstrated according to the type of infections and causative organisms.

Ueda et al. [6] reported that elevation of hepatic enzymes was frequently observed when the VRCZ concentration was >6 μg/mL. Pascual et al. [9] found that none of the patients in their series with levels ≤5.5 μg/mL presented with neurological toxicity (*P* = 0.002). Okuda et al. [10] found a significant difference (*P* < 0.05) in the average trough blood concentrations between patients presenting with adverse events and those with no adverse events (7.64 ± 2.84 vs. 1.49 ± 1.79 μg/mL). Hagiwara et al. [35] reported that the average VRCZ trough level of patients with neurological adverse events was 3.2 μg/mL and that the troughs of all the patients requiring discontinuation because of hepatic disorder were >4.0 μg/mL. Trifilio et al. [36] found that the VRCZ concentrations were correlated with aspartate aminotransferase (AST) (*P* = 0.0009) and alkaline phosphatase (ALP) (*P* = 0.03) levels. As a result of logistic analysis, the probability of hepatotoxicity at VRCZ trough concentrations of 2 and 4 μg/mL was 1.6 and 21.6 %, respectively [37]. Lutsar et al. [38] showed a 7–17 % increase in the odds of an abnormal alanine aminotransferase (ALT), AST, ALP, or bilirubin level for every 1 μg/mL increase in the plasma concentration of VRCZ. Denning et al. [39] reported that five of six patients with VRCZ concentrations >10 μg/mL developed adverse events requiring discontinuation from the study. For neurological adverse effects, the increased incidence for values >4.0 μg/mL was significant when examined in a meta-analysis (*P* = 0.02) [12]. However, many neurological adverse events have been reported to be transient and to disappear after the discontinuation of VRCZ treatment; therefore, for safety purposes, caution with regard to liver function is recommended when the trough level exceeds 4–5 μg/mL.

VRCZ exhibited nonlinear pharmacokinetics in healthy volunteers. This deviation from linear pharmacokinetics was confirmed by linearity ratios of >1 and decreasing kel (elimination rate constant) values for multiple dosing, with a consequent increase in the terminal phase *t*_1/2_ [33]. Purkins et al. [40] found that VRCZ exhibited nonlinear pharmacokinetics and that both the *C*_max_ and the area under the concentration–time curve within the dosage interval (AUC_τ_) increased disproportionately with the dose for both intravenous (IV) and oral dosing. With oral administration, patient compliance can be monitored using TDM [2, 41].

## Initial administration regimen

### Executive summary

Table 2 Initial administration regimen (B-II).

**Table 2:** Initial administration regimen of voriconazole (VRCZ)

	Loading dose (on day 1)	Maintenance dose
IV administration	6 mg/kg twice daily	3–4 mg/kg twice daily
Oral administration (between meals)		
≥40 kg	300 mg twice daily	150–200 mg twice daily
<40 kg	150 mg twice daily	100 mg twice daily^a^

^a^The oral maintenance dose can be increased to 150 mg twice daily in patients with inadequate response

### Literature review

VRCZ is available in both intravenous and oral formulations. In adults, steady-state plasma levels after intravenous infusion of 3–6 mg/kg twice daily range from 3 to 6 μg/mL [42]. Steady-state concentrations are achieved only after 5–6 days but, if a loading dose is given, earlier steady-state concentrations are obtained [39]; therefore, a loading dose of 6 mg/kg every 12 h for 2 doses in patients with intravenous administration and 300 mg every 12 h for 2 doses in oral administration is required.

Purkins et al. [43] conducted a randomized controlled trial of 12 healthy volunteers. In the trial, the bioavailability of twice-daily 200 mg VRCZ was reduced by approximately 22 %, as measured by AUC_τ_, after multiple dosing taken with food, compared with fasting. The results of this study suggest that to maximize bioavailability, VRCZ should not be administered immediately following a meal.

## TDM in patients under particular clinical conditions and pediatric considerations

### Executive summary

For patients under the following clinical conditions and for children, particular consideration is required because of unstable pharmacokinetic parameters.

- Patients with impaired renal function: As PK of VRCZ is not affected in subjects with renal impairment, no adjustment in the dosage of the oral formulation of VRCZ is necessary according to renal function (B-II). Intravenous administration is not recommended for patients with creatinine clearance <30 ml/min because of the possible accumulation of the intravenous vehicle sulfobutylether-beta-cyclodextrin (SBECD) (B-II).
- Patients undergoing dialysis: It is recommended to treat patients on dialysis therapy only with the oral form of VRCZ, if feasible. In patients to whom VRCZ is administered intravenously for any reason, possible adverse events (altered consciousness level, hemodynamic instability, skin reactions, deterioration of liver function) caused by the accumulation of SBECD should be monitored (C1-III).
- Patients with liver dysfunction: Dosage adjustments are necessary for patients with liver dysfunction. The standard loading dose should be used but the maintenance dosage should be halved in patients with mild-to-moderate liver disease (Child–Pugh Class A and B) (B-II). No studies have evaluated the safety of VRCZ in patients with severe liver disease (Child–Pugh Class C) (unresolved issue).
- Pediatric considerations
  1. Although VRCZ is not approved for pediatric patients, the dosage of 7 mg/kg every 12 h is recommended in other countries (B-II). The efficacy and safety of higher doses, including a loading dosage, are under investigation.
  2. Relatively higher hepatic clearance of VRCZ tends to cause a lower plasma concentration in pediatric patients.
  3. Lower bioavailability of oral VRCZ should be considered in pediatric patients.

### Literature review

- Patients with impaired renal function: Abel et al. [44] reported that PK of VRCZ was unaffected in subjects with any degree of renal impairment. As VRCZ has limited aqueous solubility, the intravenous form includes SBECD as a novel delivery system. In healthy subjects, SBECD is rapidly eliminated with a terminal half-life of 1.6 h. Clearance is linearly related to creatinine clearance, and accumulation has been described in subjects with moderate to severe renal impairment (serum creatinine >2.5 mg/dl). In patients with an estimated creatinine clearance of 30–50 ml/min, the mean *C*_max_ and AUC of SBECD increased by almost 50 % and fourfold, respectively, compared to subjects with normal renal function [45]. Abel et al. [44] described that clearance of SBECD was proportional to creatinine clearance. Although two subjects had >30 % increase in serum creatinine from baseline, these changes did not correlate with SBECD trough levels. The majority of subjects with moderate renal insufficiency were able to tolerate 7 days of intravenous VRCZ solubilized with SBECD. Although adverse events regarding the accumulation of SBECD have not been confirmed so far in a human study, target organs for toxic effects in rodents were the kidney and liver with obstruction of renal tubules and single-cell necrosis in the liver. Both findings were a consequence of massive cytoplasmic vacuolation [46].
- Patients undergoing dialysis: von Mach et al. [47] showed the accumulation of SBECD in patients treated with intravenous VRCZ and dialysis therapy; however, there was no evidence of toxic effects related to the concentrations of SBECD in these patients. Higher SBECD exposure seemed to be unavoidable in patients on dialysis if VRCZ was administered intravenously. Although clinically relevant toxicity with high SBECD concentrations remains unknown [48], it is recommended to treat patients on dialysis therapy only with the oral form of VRCZ, if feasible. In the study of the VRCZ plasma level in patients on dialysis, <1 % of the administered VRCZ (200 mg dose) was recovered from the dialysate 24 h after dosing in patients receiving renal replacement therapy for end-stage renal disease by peritoneal dialysis (PD). The PD clearance was 3.7 ml/min, and the VRCZ clearance was the lowest for PD, followed by continuous venovenous hemodiafiltration and hemodialysis (20 and 121 ml/min, respectively). The peritoneal clearance of VRCZ is minimal; therefore, no dosage adjustment is required for patients receiving PD therapy in whom the oral form of VRCZ is used [49].
- Patients with hepatic dysfunction: In a multiple-dose study, patients with Child–Pugh class [50] A and B liver dysfunction who received a 50 % reduced VRCZ maintenance dose achieved a mean AUC_24_ of 56.2 μg h/mL; in normal individuals who received full-dose VRCZ, the AUC_24_ was 57.8 μg h/mL. These data suggest that a 50 % reduction in the maintenance dose of VRCZ is necessary for patients with Child–Pugh Class A or B hepatic dysfunction to avoid excessive VRCZ exposure [51]. VRCZ should be administered without dosage adjustment to critically ill patients without liver cirrhosis undergoing continuous venovenous hemodiafiltration; however, according to the results in one patient, a reduction in the maintenance dosing regimen for VRCZ might be useful for patients with liver cirrhosis [52]. A VRCZ case report with Child–Pugh Class C showed that pharmacokinetic studies in patients with severe hepatic impairment should be performed to establish reliable dose recommendations for this group of patients, who are at high risk of developing invasive fungal infections. Although there is no clear evidence, we also think that several TDM are required for patients with severe hepatic impairment [53].
- Pediatrics considerations: In other countries, a dosage of 7 mg/kg every 12 h has been used [19, 20, 22, 54, 55]. The pharmacokinetics of VRCZ is highly variable in children, particularly for oral formulations [18]. Because bioavailability and clearance in children may differ from adults, a proposal for high-dose administration, including the loading dosage, is currently moving forward in Japan. Overall, a weight-based oral dose may be more appropriate than a fixed dose for children [22]. Yanni et al. [18] showed that VRCZ clearance is approximately threefold higher in children than in adults. In other reports, VRCZ clearance was higher in children than in adults [22, 56]. Walsh et al. [57] concluded that pediatric patients have a higher capacity for eliminating VRCZ than adults and that dosages of 4 mg/kg may be required for children to achieve exposure consistent with that occurring in adults after dosages of 3 mg/kg. In a pharmacokinetic analysis of children, children were especially at risk because VRCZ exhibits markedly reduced oral bioavailability in children compared with adults (44.6 vs. 96 %) [21].

## Drug–drug interactions (Table 3)

### Executive summary

**Table 3:** Drug interactions

Drug	CYP			Mechanism
	2C9	2C19	3A4	
Contraindications				
Rifampin			○	Because of induction of the CYP3A4 metabolism by rifampin, rifampin decreased the steady-state *C*_max_ and AUC of VRCZ
Rifabutin			○	Because of induction of the CYP3A4 metabolism by rifabutin, rifabutin decreased the steady-state *C*_max_ and AUC of VRCZ
				Because of inhibition of the CYP3A4 metabolism by VRCZ, VRCZ increased the steady-state *C*_max_ and AUC of rifabutin
Efavirenz	○	○	○	Because of induction of the CYP2C19 and 2C9 metabolism by efavirenz, efavirenz decreased the steady-state *C*_max_ and AUC of VRCZ
				Because of inhibition of the CYP3A4 metabolism by VRCZ, VRCZ increased the steady-state *C*_max_ and AUC of efavirenz
Ritonavir	○	○		Because of induction of the CYP2C19 and 2C9 metabolism by ritonavir, ritonavir decreased the steady-state *C*_max_ and AUC of VRCZ
Carbamazepine			○	Because of induction of the CYP3A4 metabolism by carbamazepine, carbamazepine decreased the steady-state *C*_max_ and AUC of VRCZ
Barbital			○	Because of induction of the CYP3A4 metabolism by barbital, barbital decreased the steady-state *C*_max_ and AUC of VRCZ
Phenobarbital			○	Because of induction of the CYP3A4 metabolism by phenobarbital, phenobarbital decreased the steady-state *C*_max_ and AUC of VRCZ
Pimozide			○	Because of inhibition of the CYP3A4 metabolism by VRCZ, VRCZ increased plasma concentration and risk of cardiotoxicity (QT prolongation, torsade de pointes, cardiac arrest) of pimozide
Quinidine			○	Because of inhibition of the CYP3A4 metabolism by VRCZ, VRCZ increased plasma concentration and risk of cardiotoxicity (QT prolongation, torsade de pointes, cardiac arrest) of quinidine
Ergotamine			○	Because of inhibition of the CYP3A4 metabolism by VRCZ, VRCZ increased plasma concentration of ergot derivative and an increased risk of ergotism (nausea, vomiting, vasospastic ischemia) of ergotamine
Triazolam			○	Because of inhibition of the CYP3A4 triazolam metabolism by VRCZ, VRCZ increased plasma concentrations and potential of triazolam
Cautions				
Phenytoin	○		○	Because of induction of the CYP3A4 metabolism by phenytoin, phenytoin decreased the steady-state *C*_max_ and AUC of VRCZ
				Because of inhibition of the CYP2C9 metabolism by VRCZ, VRCZ increased the steady-state *C*_max_ and AUC of phenytoin
Inhibitor of HIV protease (excluded indinavir): saquinavir, amprenavir, nelfinavir			○	Because of inhibition of the CYP3A4 metabolism by VRCZ, VRCZ increased plasma concentration of HIV protease inhibitor
				Because of inhibition of the CYP3A4 metabolism by HIV protease inhibitor, HIV protease inhibitor increased plasma concentration of VRCZ
(n*) nucleoside reverse transcriptase inhibitor (NNRTI): Delavirdine			○	Because of inhibition of the CYP3A4 metabolism by NNRTI, NNRTI increased plasma concentration of VRCZ
				Because of induction of the CYP3A4 metabolism by NNRT, NNRT decreased plasma concentration of VRCZ
				Because of inhibition of the CYP3A4 metabolism by VRCZ VRCZ increased plasma concentration of NNRTI
Cyclosporine			○	Because of inhibition of the CYP3A4 metabolism by VRCZ, VRCZ increased the steady-state *C*_max_ and AUC of cyclosporine
Tacrolimus			○	Because of inhibition of the CYP3A4 metabolism by VRCZ, VRCZ increased the steady-state *C*_max_ and AUC of tacrolimus
Warfarin	○			Because of inhibition of the CYP2C9 metabolism by VRCZ, VRCZ increased the prothrombin time of warfarin
Omeprazole		○	○	Because of inhibition of the CYP2C19 and 3A4 metabolism by VRCZ, VRCZ increased the steady-state *C*_max_ and AUC of omeprazole
Midazolam			○	Because of inhibition of the CYP3A4 metabolism by VRCZ, VRCZ increased plasma concentration of midazolam
HMG-CoA reductase inhibitor			○	Because of inhibition of the CYP3A4 metabolism by VRCZ, VRCZ increased plasma concentration of HMG-CoA reductase inhibitor
Diazepam	○		○	Because of inhibition of the CYP2C9 and 3A4 metabolism by VRCZ, VRCZ increased the steady-state AUC and elimination half-life of diazepam
Zolpidem	○		○	Because of inhibition of the CYP2C9 and 3A4 metabolism by VRCZ, VRCZ increased the steady-state *C*_max_ and AUC of zolpidem
Sulfonylureas; tolbutamide	○			Because of inhibition of the CYP2C9 metabolism by VRCZ, VRCZ increased plasma concentration of sulfonylureas
Vinca alkaloids anticancer agents			○	Because of inhibition of the CYP3A4 metabolism by VRCZ, VRCZ increased plasma concentration of vinca alkaloids
Vincristine				
Vinblastine				
Oxycodone			○	Because of inhibition of the CYP3A4 metabolism by VRCZ, VRCZ increased the steady-state *C*_max_ and AUC of oxycodone
Fentanyl			○	Because of inhibition of the CYP3A4 metabolism by VRCZ, VRCZ increased the steady-state AUC of fentanyl
Ibuprofen	○			Because of inhibition of the CYP2C9 metabolism by VRCZ, VRCZ increased steady-state *C*_max_ and AUC of ibuprofen
Diclofenac	○			Because of inhibition of the CYP2C9 metabolism by VRCZ, VRCZ increased steady-state *C*_max_ and AUC of diclofenac
Oral contraceptive; norethindrone and ethinyl estradiol		○	○	Because of inhibition of the CYP2C19 metabolism by norethindrone and ethinyl estradiol, norethindrone and ethinyl estradiol increased steady-state *C*_max_ and AUC of VRCZ
				Because of inhibition of the CYP3A4 metabolism by VRCZ, VRCZ increased steady-state *C*_max_ and AUC of norethindrone and ethinyl estradiol
St. John’s wort		○	○	Because of induction of the CYP3A4 and 2C19 metabolism by St. John’s wort, St. John’s wort decreased the steady-state AUC of VRCZ
Overseas reference				
Sirolimus			○	Because of inhibition of the CYP3A4 metabolism by VRCZ, VRCZ increased the steady-state *C*_max_ and AUC of sirolimus
Digoxin, cimetidine, ranitidine				No change

- VRCZ has an inhibitory effect on CYP2C19, 2C9, and 3A4; therefore, careful attention is required when drugs that are metabolized by these metabolizing enzymes are administered concurrently with VRCZ (B-II).
- Serious consideration should be given to raising the blood concentration of calcineurin inhibitors by two- to threefold when VRCZ and calcineurin inhibitors are coadministered (B-II).

### Literature review

Mikus et al. [28] reported that the coadministration of a potent CYP3A4 inhibitor leads to higher and prolonged exposure to VRCZ, possibly increasing the risk of adverse drug reactions. In a trial to study the interactions between VRCZ and omeprazole, omeprazole was found to have no clinically relevant effect on VRCZ exposure, suggesting that no VRCZ dosage adjustment is necessary for patients in whom omeprazole therapy is initiated [58]. Purkins et al. [59] conducted two studies pertinent to the interactions between VRCZ and phenytoin. Consequently, repeated dose administration of phenytoin was shown to decrease the mean steady-state *C*_max_ and AUC_τ_ of VRCZ by approximately 50 and 70 %, respectively. The repeated dose administration of 400 mg VRCZ twice daily increased the mean steady-state *C*_max_ and AUC_τ_ of phenytoin by approximately 70  and 80 %, respectively. Consequently, the plasma phenytoin concentrations should be monitored.

Takashima et al. [60] conducted a retrospective study of HSCT patients treated with tacrolimus. The oral administration of VRCZ increased the blood concentration/dose (C/D) ratio of tacrolimus by 2.7 fold, and the IV administration of VRCZ increased the C/D ratio by 2 fold. Considering these results, the dosages of calcineurin inhibitors should be reduced by 50–65 % when concomitantly administered with VRCZ. Trifilio et al. [61] suggested that the dosage of tacrolimus may need to be reduced by 30–40 %. When VRCZ and calcineurin inhibitors are coadministered, close and periodic monitoring of the tacrolimus and cyclosporine concentrations is necessary in each patient to minimize dose-related toxicity and to maximize efficacy [62, 63].

## Measurement of blood concentrations

### Executive summary

High performance liquid chromatography (HPLC) is recommended to measure blood concentrations. No factors affecting such measurements have been reported (B-III).

### Literature review

To perform TDM, a validated analytical method must be available to determine VRCZ plasma concentrations. The various methods employed include HPLC with ultraviolet or mass spectrometric and bioassays; however, for the treatment of invasive antifungal infection, bioassays lack sensitivity and specificity compared with HPLC, when combination therapy is being used. Thus, HPLC may be the most suitable assay for measuring VRCZ concentrations [64–83].

## References

[1] Cecil JA, Wenzel RP (2009). Voriconazole: a broad-spectrum triazole for the treatment of invasive fungal infections. Expert Rev Hematol.

[2] Brüggemann RJ, Donnelly JP, Aarnoutse RE, Warris A, Blijlevens NM, Mouton JW (2008). Therapeutic drug monitoring of voriconazole. Ther Drug Monit.

[3] Canadian Task Force on the Periodic Health Examination (1979). The Periodic Health Examination. Can Med Assoc J.

[4] Tan K, Brayshaw N, Tomaszewski K, Troke P, Wood N (2006). Investigation of the potential relationships between plasma voriconazole concentrations and visual adverse events or liver function test abnormalities. J Clin Pharmacol.

[5] Kuo IF, Ensom MHH (2009). Role of therapeutic drug monitoring of voriconazole in the treatment of invasive fungal infections. Can J Hosp Pharm.

[6] Ueda K, Nannya Y, Kumano K, Hangaishi A, Takahashi T, Imai Y (2009). Monitoring trough concentration of voriconazole is important to ensure successful antifungal therapy and to avoid hepatic damage in patients with hematological disorders. Int J Hematol.

[7] Trifilio S, Singhal S, Williams S, Frankfurt O, Gordon L, Evens A (2007). Breakthrough fungal infections after allogeneic hematopoietic stem cell transplantation in patients on prophylactic voriconazole. Bone Marrow Transpl.

[8] Smith J, Safdar N, Knasinski V, Simmons W, Bhavnani SM, Ambrose PG (2006). Voriconazole therapeutic drug monitoring. Antimicrob Agents Chemother.

[9] Pascual A, Calandra T, Bolay S, Buclin T, Bille J, Marchetti O (2008). Voriconazole therapeutic drug monitoring in patients with invasive mycoses improves efficacy and safety outcomes. Clin Infect Dis.

[10] Okuda T, Okuda A, Watanabe N, Takao M, Takayanagi K (2008). Retrospective serological tests for determining the optimal blood concentration of voriconazole for treating fungal infection. Yakugaku Zasshi.

[11] Miyakis S, van Hal SJ, Ray J, Marriott D (2010). Voriconazole concentrations and outcome of invasive fungal infections. Clin Microbiol Infect.

[12] Hamada Y, Seto Y, Yago K, Kuroyama M (2012). Investigation and threshold of optimum blood concentration of voriconazole: a descriptive statistical meta-analysis. J Infect Chemother.

[13] Trifilio S, Pennick G, Pi J, Zook J, Golf M, Kaniecki K (2007). Monitoring plasma voriconazole levels may be necessary to avoid subtherapeutic levels in hematopoietic stem cell transplant recipients. Cancer (Phila).

[14] Troke PF, Hockey HP, Hope WW (2011). Observational study of the clinical efficacy of voriconazole and its relationship to plasma concentrations in patients. Antimicrob Agents Chemother.

[15] Levin MD, den Hollander JG, van der Holt B, Rijnders BJ, van Vliet M, Sonneveld P (2007). Hepatotoxicity of oral and intravenous voriconazole in relation to cytochrome P450 polymorphisms. J Antimicrob Chemother.

[16] Kakuda TN, Van Solingen-Ristea R, Aharchi F, De Smedt G, Witek J, Nijs S, et al. Pharmacokinetics and short-term safety of etravirine in combination with fluconazole or voriconazole in HIV-negative volunteers. J Clin Pharmacol. 2012 [Epub ahead of print]. 10.1177/0091270011433329 23400742

[17] Park WB, Kim NH, Kim KH, Lee SH, Nam WS, Yoon SH (2012). The effect of therapeutic drug monitoring on safety and efficacy of voriconazole in invasive fungal infections: a randomized controlled trial. Clin Infect Dis.

[18] Yanni SB, Annaert PP, Augustijns P, Ibrahim JG, Benjamin DK, Thakker DR (2010). In vitro hepatic metabolism explains higher clearance of voriconazole in children versus adults: role of CYP2C19 and flavin-containing monooxygenase 3. Drug Metab Dispos.

[19] Neely M, Rushing T, Kovacs A, Jelliffe R, Hoffman J (2010). Voriconazole pharmacokinetics and pharmacodynamics in children. Clin Infect Dis.

[20] Michael C, Bierbach U, Frenzel K, Lange T, Basara N, Niederwieser D (2010). Voriconazole pharmacokinetics and safety in immunocompromised children compared to adult patients. Antimicrob Agents Chemother.

[21] Karlsson MO, Lutsar I, Milligan PA (2009). Population pharmacokinetic analysis of voriconazole plasma concentration data from pediatric studies. Antimicrob Agents Chemother.

[22] Driscoll TA, Yu LC, Frangoul H, Krance RA, Nemecek E, Blumer J (2011). Comparison of pharmacokinetics and safety of voriconazole intravenous-to-oral switch in immunocompromised children and healthy adults. Antimicrob Agents Chemother.

[23] Driscoll TA, Frangoul H, Nemecek ER, Murphey DK, Yu LC, Blumer J (2011). Comparison of pharmacokinetics and safety of voriconazole intravenous-to-oral switch in immunocompromised adolescents and healthy adults. Antimicrob Agents Chemother.

[24] Trifilio SM, Yarnold PR, Scheetz MH, Pi J, Pennick G, Mehta J (2009). Serial plasma voriconazole concentrations after allogeneic hematopoietic stem cell transplantation. Antimicrob Agents Chemother.

[25] Andes D, Marchillo K, Stamstad T, Conklin R (2003). In vivo pharmacokinetics and pharmacodynamics of a new triazole, voriconazole, in a murine candidiasis model. Antimicrob Agents Chemother.

[26] Andes D (2003). Pharmacokinetics and pharmacodynamics in the development of antifungal compounds. Curr Opin Invest Drugs.

[27] Johnson LB, Kauffman CA (2003). Voriconazole: a new triazole antifungal agent. Clin Infect Dis.

[28] Mikus G, Schowel V, Drzewinska M, Rengelshausen J, Ding R, Riedel KD (2006). Potent cytochrome P450 2C19 genotype-related interaction between voriconazole and the cytochrome P450 3A4 inhibitor ritonavir. Clin Pharmacol Ther.

[29] Kubota T, Chiba K, Ishizaki T (1996). Genotyping of *S*-mephenytoin 4′-hydroxylation in an extended Japanese population. Clin Pharmacol Ther.

[30] Hyland R, Jones BC, Smith DA (2003). Identification of the cytochrome P450 enzymes involved in the N-oxidation of voriconazole. Drug Metab Dispos.

[31] Desta Z, Zhao X, Shin JG, Flockhart DA (2002). Clinical significance of the cytochrome P450 2C19 genetic polymorphism. Clin Pharmacokinet.

[32] Kimura M, Yamagishi Y, Kawasumi N, Hagihara M, Hasegawa T, Mikamo H (2010). Clinical implication of therapeutic drug monitoring on voriconazole from the aspect of the analysis for CYP2C19 gene. Jpn J Antibiot.

[33] Purkins L, Wood N, Greenhalgh K, Allen MJ, Oliver SD (2003). Voriconazole, a novel wide-spectrum triazole: oral pharmacokinetics and safety. Br J Clin Pharmacol.

[34] Lazarus HM, Blumer JL, Yanovich S, Schlamm H, Romero A (2002). Safety and pharmacokinetics of oral voriconazole in patients at risk of fungal infection: a dose escalation study. J Clin Pharmacol.

[35] Hagiwara E, Shiihara J, Matsushima A, Enomoto T, Tagawa A, Sekine A (2009). Usefulness of monitoring plasma voriconazole concentration in patients with chronic necrotizing pulmonary aspergillosis. Nihon Kokyuki Gakkai Zasshi.

[36] Trifilio S, Ortiz R, Pennick G, Verma A, Pi J, Stosor V (2005). Voriconazole therapeutic drug monitoring in allogeneic hematopoietic stem cell transplant recipients. Bone Marrow Transpl.

[37] Matsumoto K, Ikawa K, Abematsu K, Fukunaga N, Nishida K, Fukamizu T (2009). Correlation between voriconazole trough plasma concentration and hepatotoxicity in patients with different CYP2C19 genotypes. Int J Antimicrob Agents.

[38] Lutsar I, Hodges MR, Tomaszewski K, Troke PF, Wood ND (2003). Safety of voriconazole and dose individualization. Clin Infect Dis.

[39] Denning DW, Ribaud P, Milpied N, Caillot D, Herbrecht R, Thiel E (2002). Efficacy and safety of voriconazole in the treatment of acute invasive aspergillosis. Clin Infect Dis.

[40] Purkins L, Wood N, Ghahramani P, Greenhalgh K, Allen MJ, Kleinermans D (2002). Pharmacokinetics and safety of voriconazole following intravenous- to oral-dose escalation regimens. Antimicrob Agents Chemother.

[41] Hassan A, Burhenne J, Riedel KD, Weiss J, Mikus G, Haefeli WE (2011). Modulators of very low voriconazole concentrations in routine therapeutic drug monitoring. Ther Drug Monit.

[42] Hoffman HL, Rathbun RC (2002). Review of the safety and efficacy of voriconazole. Expert Opin Invest Drugs.

[43] Purkins L, Wood N, Kleinermans D, Greenhalgh K, Nichols D (2003). Effect of food on the pharmacokinetics of multiple-dose oral voriconazole. Br J Clin Pharmacol.

[44] Abel S, Allan R, Gandelman K, Tomaszewski K, Webb DJ, Wood ND (2008). Pharmacokinetics, safety and tolerance of voriconazole in renally impaired subjects: two prospective, multicentre, open-label, parallel-group volunteer studies. Clin Drug Invest.

[45] FDA Antiviral Drugs Advisory Committee—Briefing document for voriconazole (oral and intravenous formulations). http://www.fda.gov/ohrms/dockets/ac/01/briefing/3792b2_01_Pfizer.pdf.

[46] Background Document for the Antiviral Drug Products Advisory Committee Meeting. http://www.fda.gov/ohrms/dockets/ac/01/briefing/3792b2_02_FDA-voriconazole.htm.

[47] von Mach MA, Burhenne J, Weilemann LS (2006). Accumulation of the solvent vehicle sulphobutylether beta cyclodextrin sodium in critically ill patients treated with intravenous voriconazole under renal replacement therapy. BMC Clin Pharmacol.

[48] Hafner V, Czock D, Burhenne J, Riedel KD, Bommer J, Mikus G (2010). Pharmacokinetics of sulfobutylether-beta-cyclodextrin and voriconazole in patients with end-stage renal failure during treatment with two hemodialysis systems and hemodiafiltration. Antimicrob Agents Chemother.

[49] Peng LW, Lien YH (2005). Pharmacokinetics of single, oral-dose voriconazole in peritoneal dialysis patients. Am J Kidney Dis.

[50] Pugh RN, Murray-Lyon IM, Dawson JL, Pietroni MC, Williams R (1973). Transection of the oesophagus for bleeding oesophageal varices. Br J Surg.

[51] Cota JM, Burgess DS (2010). Antifungal dose adjustment in renal and hepatic dysfunction: pharmacokinetic and pharmacodynamic considerations. Curr Fungal Infect Rep.

[52] Fuhrmann V, Schenk P, Jaeger W, Miksits M, Kneidinger N, Warszawska J (2007). Pharmacokinetics of voriconazole during continuous venovenous haemodiafiltration. J Antimicrob Chemother.

[53] Weiler S, Zoller H, Graziadei I, Vogel W, Bellmann-Weiler R, Joannidis M (2007). Altered pharmacokinetics of voriconazole in a patient with liver cirrhosis. Antimicrob Agents Chemother.

[54] Goutelle S, Larcher R, Padoin C, Mialou V, Bleyzac N (2010). Oral voriconazole dose in children: one size does not fit all. Clin Infect Dis.

[55] Bruggemann RJ, van der Linden JW, Verweij PE, Burger DM, Warris A (2011). Impact of therapeutic drug monitoring of voriconazole in a pediatric population. Pediatr Infect Dis J.

[56] Ito S, Ishizaki J, Koshiba M, Igarashi Y, Nagata N, Suga Y (2010). Comparison of relationship between dosage and serum concentration of voriconazole in adult and pediatric patients. Iryoyakugaku.

[57] Walsh TJ, Karlsson MO, Driscoll T, Arguedas AG, Adamson P, Saez-Llorens X (2004). Pharmacokinetics and safety of intravenous voriconazole in children after single- or multiple-dose administration. Antimicrob Agents Chemother.

[58] Wood N, Tan K, Purkins L, Layton G, Hamlin J, Kleinermans D (2003). Effect of omeprazole on the steady-state pharmacokinetics of voriconazole. Br J Clin Pharmacol.

[59] Purkins L, Wood N, Ghahramani P, Love ER, Eve MD, Fielding A (2003). Coadministration of voriconazole and phenytoin: pharmacokinetic interaction, safety, and toleration. Br J Clin Pharmacol.

[60] Takashima M, Taniguchi R, Yano L, Kono T, Hashida T, Masuda S (2009). Pharmacokinetic interactions between calcineurin inhibitors and azole antifungals in hematopoietic stem cell transplant recipients. Jpn J Pharm Health Care Sci.

[61] Trifilio SM, Scheetz MH, Pi J, Mehta J (2010). Tacrolimus use in adult allogeneic stem cell transplant recipients receiving voriconazole: preemptive dose modification and therapeutic drug monitoring. Bone Marrow Transpl.

[62] Mori T, Aisa Y, Kato J, Nakamura Y, Ikeda Y, Okamoto S (2009). Drug interaction between voriconazole and calcineurin inhibitors in allogeneic hematopoietic stem cell transplant recipients. Bone Marrow Transpl.

[63] Kawazoe H, Takiguchi Y, Tanaka H, Fukuoka N, Ohnishi H, Ishida T (2006). Change of the blood concentration of tacrolimus after the switch from fluconazole to voriconazole in patients receiving allogeneic hematopoietic stem cell transplantation. Biol Pharm Bull.

[64] Xiong X, Duan J, Zhai S, Wang L, Lan X (2010). Fast and reliable determination of voriconazole in human plasma by LC-APCI-MS/MS. Biosci Biotechnol Biochem.

[65] Wu XJ, Dong XY, Chen YJ, Zhang J, Shi YG, Wu JF (2009). A HPLC method for voriconazole therapeutic drug monitoring. Chin J Infect Chemother.

[66] Wenk M, Droll A, Krahenbuhl S (2006). Fast and reliable determination of the antifungal drug voriconazole in plasma using monolithic silica rod liquid chromatography. J Chromatogr B Anal Technol Biomed Life Sci.

[67] Vogeser M, Schiel X, Spohrer U (2005). Quantification of voriconazole in plasma by liquid chromatography-tandem mass spectrometry. Clin Chem Lab Med.

[68] Vogeser M (2005). The use of HPLC-tandem mass spectrometry in therapeutic drug monitoring. Laboratoriums Medizin.

[69] Verdier MC, Bentue-Ferrer D, Tribut O, Bellissant E (2010). Liquid chromatography–tandem mass spectrometry method for simultaneous quantification of four triazole antifungal agents in human plasma. Clin Chem Lab Med.

[70] Theurillat R, Zimmerli S, Thormann W (2010). Determination of voriconazole in human serum and plasma by micellar electrokinetic chromatography. J Pharm Biomed Anal.

[71] Spriet I, Annaert P, Meersseman P, Hermans G, Meersseman W, Verbesselt R (2009). Pharmacokinetics of caspofungin and voriconazole in critically ill patients during extracorporeal membrane oxygenation. J Antimicrob Chemother.

[72] Perea S, Pennick GJ, Modak A, Fothergill AW, Sutton DA, Sheehan DJ (2000). Comparison of high-performance liquid chromatographic and microbiological methods for determination of voriconazole levels in plasma. Antimicrob Agents Chemother.

[73] Pehourcq F, Jarry C, Bannwarth B (2004). Direct injection HPLC micro method for the determination of voriconazole in plasma using an internal surface reversed-phase column. Biomed Chromatogr.

[74] Pascual A, Nieth V, Calandra T, Bille J, Bolay S, Decosterd LA (2007). Variability of voriconazole plasma levels measured by new high-performance liquid chromatography and bioassay methods. Antimicrob Agents Chemother.

[75] Nakagawa S, Suzuki R, Yamazaki R, Kusuhara Y, Mitsumoto S, Kobayashi H (2008). Determination of the antifungal agent voriconazole in human plasma using a simple column-switching high-performance liquid chromatography and its application to a pharmacokinetic study. Chem Pharm Bull (Tokyo).

[76] Michael C, Bierbach U, Frenzel K, Lange T, Basara N, Niederwieser D (2010). Determination of saliva trough levels for monitoring voriconazole therapy in immunocompromised children and adults. Ther Drug Monit.

[77] Lin SC, Lin SW, Chen JM, Kuo CH (2010). Using sweeping-micellar electrokinetic chromatography to determine voriconazole in patient plasma. Talanta.

[78] Gordien JB, Pigneux A, Vigouroux S, Tabrizi R, Accoceberry I, Bernadou JM (2009). Simultaneous determination of five systemic azoles in plasma by high-performance liquid chromatography with ultraviolet detection. J Pharm Biomed Anal.

[79] Farowski F, Cornely OA, Vehreschild JJ, Hartmann P, Bauer T, Steinbach A (2010). Quantitation of azoles and echinocandins in compartments of peripheral blood by liquid chromatography–tandem mass spectrometry. Antimicrob Agents Chemother.

[80] Decosterd LA, Rochat B, Pesse B, Mercier T, Tissot F, Widmer N (2010). Multiplex ultra-performance liquid chromatography–tandem mass spectrometry method for simultaneous quantification in human plasma of fluconazole, itraconazole, hydroxyitraconazole, posaconazole, voriconazole, voriconazole-*N*-oxide, anidulafungin, and caspofungin. Antimicrob Agents Chemother.

[81] Chhun S, Rey E, Tran A, Lortholary O, Pons G, Jullien V (2007). Simultaneous quantification of voriconazole and posaconazole in human plasma by high-performance liquid chromatography with ultra-violet detection. J Chromatogr B Anal Technol Biomed Life Sci.

[82] Chahbouni A, Wilhelm AJ, den Burger JC, Sinjewel A, Vos RM (2010). Validated liquid chromatography–tandem mass spectroscopy method for the simultaneous quantification of four antimycotic agents in human serum. Ther Drug Monit.

[83] Alffenaar JW, Wessels AM, van Hateren K, Greijdanus B, Kosterink JG, Uges DR (2010). Method for therapeutic drug monitoring of azole antifungal drugs in human serum using LC/MS/MS. J Chromatogr B Anal Technol Biomed Life Sci.

